# Randomized, double‐blind, phase two study of ruxolitinib plus regorafenib in patients with relapsed/refractory metastatic colorectal cancer

**DOI:** 10.1002/cam4.1703

**Published:** 2018-08-19

**Authors:** David Fogelman, Antonio Cubillo, Pilar García‐Alfonso, María Luisa Limón Mirón, John Nemunaitis, Daniel Flora, Christophe Borg, Laurent Mineur, Jose M. Vieitez, Allen Cohn, Gene Saylors, Albert Assad, Julie Switzky, Li Zhou, Johanna Bendell

**Affiliations:** ^1^ The University of Texas MD Anderson Cancer Center Houston Texas; ^2^ Centro Integral Oncológico Clara Campal Madrid Spain; ^3^ Hospital General Universitario Gregorio Marañón Madrid Spain; ^4^ Hospital Universitario Virgen del Rocío Sevilla Spain; ^5^ University of Toledo College of Medicine and Life Sciences Toledo Ohio; ^6^ Oncology Hematology Care Cincinnati Ohio; ^7^ University Hospital of Besançon Besançon France; ^8^ Institut Sainte Catherine Avignon France; ^9^ Hospital Universitario Central de Asturias Oviedo Spain; ^10^ Rocky Mountain Cancer Centers Denver Colorado; ^11^ Charleston Hematology Oncology Associates Charleston South Carolina; ^12^ Incyte Corporation Wilmington, Delaware; ^13^ Sarah Cannon Research Institute/Tennessee Oncology Nashville Tennessee

**Keywords:** clinical trial, colorectal cancer, inflammation, JAK1 protein tyrosine kinase, JAK2 protein tyrosine kinase, ruxolitinib

## Abstract

**Background:**

The Janus kinase/signal transducer and activator of transcription (JAK‐STAT) signaling pathway plays a key role in the systemic inflammatory response in many cancers, including colorectal cancer (CRC). This study evaluated the addition of ruxolitinib, a potent JAK1/2 inhibitor, to regorafenib in patients with relapsed/refractory metastatic CRC.

**Methods:**

In this two‐part, multicenter, phase 2 study, eligible adult patients had metastatic adenocarcinoma of the colon or rectum; an Eastern Cooperative Oncology Group performance status of 0‐2; received fluoropyrimidine, oxaliplatin, and irinotecan‐based chemotherapy, an anti‐vascular endothelial growth factor therapy (if no contraindication); and if *KRAS* wild‐type (and no contraindication), an anti‐epidermal growth factor receptor therapy; and progressed following the last administration of approved therapy. Patients who received previous treatment with regorafenib, had an established cardiac or gastrointestinal disease, or had an active infection requiring treatment were excluded. The study was conducted in 95 sites in North America, European Union, Asia Pacific, and Israel. After an open‐label, safety run‐in phase (part 1; ruxolitinib 20 mg twice daily [BID] plus regorafenib 160 mg once daily [QD]), the double‐blind, randomized phase (part 2) was conducted wherein patients were randomized 1:1 to receive ruxolitinib 15 mg BID plus regorafenib 160 mg QD [ruxolitinib group] or placebo plus regorafenib 160 mg QD [placebo group]. Part 2 included substudy 1 (patients with high systemic inflammation, ie, C‐reactive protein [CRP] >10 mg/L) and substudy 2 (patients with low systemic inflammation, ie, CRP ≤10 mg/L); the primary endpoint was overall survival (OS).

**Results:**

The study was terminated early; substudy 1 was terminated for futility at interim analysis and substudy 2 was terminated per sponsor decision. Ruxolitinib 20 mg BID was well tolerated in the safety run‐in (n = 11). Overall, 396 patients were randomized (substudy 1: n = 175 [ruxolitinib group, n = 87; placebo group, n = 88]; substudy 2: n = 221 [ruxolitinib group, n = 110; placebo group, n = 111]). There was no significant difference in OS or progression‐free survival (PFS) between treatments in substudy 1 (OS: hazard ratio [HR] = 1.040 [95% confidence interval: 0.725‐1.492]; PFS: HR = 1.004 [0.724‐1.391]) and substudy 2 (OS: HR = 0.767 [0.478‐1.231]; PFS: HR = 0.787 [0.576‐1.074]). The most common hematologic adverse event was anemia. No new safety signals with ruxolitinib were identified.

**Conclusions:**

Although addition of ruxolitinib to regorafenib did not show increased safety concerns in patients with relapsed/refractory metastatic CRC, this combination did not improve OS/PFS vs. regorafenib plus placebo.

## 1 INTRODUCTION

Colorectal cancer (CRC) induces a systemic inflammatory response, possibly from proinflammatory cytokine production by colorectal tumor cells, the tumor microenvironment, or both.^1^ Systemic inflammatory response, as measured by markers including elevated C‐reactive protein (CRP), is linked to the poor prognosis of CRC.^1^ Evidence shows that the Janus kinase/signal transducer and activator of transcription (JAK/STAT) signaling pathway plays a role in the systemic inflammatory response in CRC.^1^, ^2^, ^3^, ^4^, ^5^, ^6^

Ruxolitinib, an oral selective inhibitor of JAK1/JAK2, is approved by the United States Food and Drug Administration and the European Medicines Agency for adult patients with intermediate‐risk or high‐risk myelofibrosis, and for patients with polycythemia vera who have an inadequate response to or are intolerant of hydroxyurea.^7^, ^8^ Of note, a subgroup analysis of the randomized, phase 2 RECAP study suggested a survival benefit with ruxolitinib in combination with capecitabine vs. capecitabine alone, in patients with metastatic pancreatic cancer and high levels of systemic inflammatory response.^9^

Regorafenib is an oral multi‐targeted kinase inhibitor that targets angiogenic, stromal, and oncogenic receptor tyrosine kinases.^10^ Regorafenib has demonstrated improved survival in patients with metastatic CRC (mCRC) who have progressed after all standard therapies^11^ and is approved for treatment of patients with refractory mCRC.

We hypothesized that the combination of ruxolitinib and regorafenib could represent a novel approach to reprogram the tumor microenvironment by simultaneously targeting inflammatory cells associated with colorectal carcinogenesis, such as myeloid‐derived suppressor cells or regulatory T cells, along with angiogenesis.^12^ Regorafenib also does not cause cytopenias to the degree that is associated with conventional chemotherapy regimens, and the nonhematologic toxicity profiles of regorafenib and ruxolitinib generally do not overlap.^13^ Thus, we designed a 2‐part phase 2 study to assess whether the addition of ruxolitinib to regorafenib would be safe and would increase the efficacy of regorafenib in patients with relapsed/refractory mCRC.

## 2 METHODS

### 2.1 Study design

The open‐label, safety run‐in phase (part 1) verified the safety of the selected doses of ruxolitinib and regorafenib. Part 2 included 2 double‐blind, randomized substudies targeting separate populations of patients with mCRC based on levels of systemic inflammation, as measured by the modified Glasgow Prognostic Score (mGPS)^14^ (substudy 1: mGPS 1 [CRP >10 mg/L and albumin ≥35 g/dL] or mGPS 2 [CRP >10 mg/L and albumin <35 mg/dL]; substudy 2: mGPS 0 [CRP ≤ 10 mg/L]). Ninety‐five sites in North America (NA) and the rest of the world (ROW; included the European Union, Asia Pacific, and Israel) participated in this study, which was approved by each site's ethics review board. The study was conducted in accordance with the Declaration of Helsinki, Good Clinical Practice guidelines, and applicable local regulations. All patients provided written informed consent before study participation. The study was registered at http://www.clinicaltrials.gov (NCT02119676).

### 2.2 Study population

Adult patients with radiographically measurable/evaluable metastatic adenocarcinoma of the colon or rectum (per Response Evaluation Criteria In Solid Tumors version 1.1) and an Eastern Cooperative Oncology Group performance status (ECOG PS) of 0‐2 were enrolled. Patients should have received previous treatment with fluoropyrimidine‐based, oxaliplatin‐based, and irinotecan‐based chemotherapy; an anti‐vascular endothelial growth factor therapy (if no contraindication); and if Kirsten Rat Sarcoma wild type (and no contraindication), an anti‐epidermal growth factor receptor therapy; and progressed following the last administration of approved therapy. Patients who received previous treatment with regorafenib, had an established cardiac or gastrointestinal disease, or had an active infection requiring treatment were excluded.

### 2.3 Randomization

Patients enrolled in part 1 were not randomized. Patients enrolled in each of the 2 substudies in part 2 were randomized (1:1) centrally by an interactive response technology system to receive ruxolitinib plus regorafenib (ruxolitinib group) or placebo plus regorafenib (placebo group). Randomization in substudy 1 was stratified by mGPS status (1 vs. 2) and geographical region (NA vs. ROW), and in substudy 2 by geographical region.

### 2.4 Treatment

**Figure 1:**
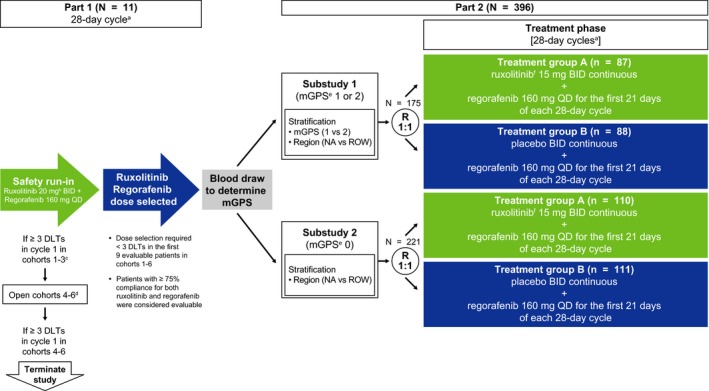
Study design. ^a^Treatment cycles continued as long as the regimen is tolerated and the patient does not meet the discontinuation criteria. ^b^Ruxolitinib starting dose was 20 mg, but it could potentially be reduced to 15 mg and 10 mg. ^c^Cohorts 1‐3: 160 mg regorafenib + 20 mg ruxolitinib (cohort 1), 15 mg ruxolitinib (cohort 2), or 10 mg ruxolitinib (cohort 3). ^d^Cohorts 4‐6: 120 mg regorafenib + 20 mg ruxolitinib (cohort 1), 15 mg ruxolitinib (cohort 2), or 10 mg ruxolitinib (cohort 3). ^e^mGPS 0: CRP ≤10 mg/L; mGPS 1: CRP >10 mg/L and albumin ≥35 g/L; mGPS 2: CRP >10 mg/L and albumin < 35 g/L. ^f^Patients who have stable laboratory parameters for neutrophils and platelets (i.e., ≤ grade 1) for 2 complete cycles will be eligible for an increase in the dose of ruxolitinib (maximum dose 20 mg BID). Abbreviations: BID; twice daily; CRP, C‐reactive protein; DLTs, dose‐limiting toxicities; mGPS, modified Glasgow Prognostic Score; NA, North America; QD, once daily; R, randomization; ROW, rest of world

Patients in part 1 (1‐6 potential cohorts of up to 9 patients each) received open‐label ruxolitinib and regorafenib (Figure 1). In cohort 1, regorafenib (160 mg once daily [QD]) was administered for the first 21 days and ruxolitinib (20 mg twice daily [BID]) for the entire 28‐day cycle. Planned enrollment into subsequent cohorts at lower doses would be based on the occurrence of ≥3 protocol‐defined dose‐limiting toxicities (DLTs) in a given cohort during cycle 1.

If ruxolitinib 20 mg BID was deemed to be tolerated in part 1, the starting dose of ruxolitinib in part 2 would be 15 mg BID with titration to 20 mg BID allowed in patients who tolerated the combination. If ruxolitinib 15 or 10 mg BID was determined to be tolerated in part 1, treatment in part 2 would begin with these doses, respectively, and titration to higher doses of ruxolitinib would not be allowed.

In part 2, patients within substudies 1 and 2 were randomized to the ruxolitinib or placebo groups at doses determined in part 1. All treatments were oral, self‐administered, and consisted of repeating 28‐day cycles.

### 2.5 Assessments

The primary objective of part 1 was to determine safe and tolerable doses of ruxolitinib and regorafenib when administered in combination, with safety and tolerability being the primary endpoint. The primary objective of part 2 was to evaluate and compare overall survival (OS; primary endpoint; defined as time from randomization to death due to any cause) between the ruxolitinib and placebo groups. Secondary endpoints (part 2 only) included progression‐free survival (PFS; defined as time from randomization to the earlier of death or disease progression), objective response rate (ORR), and safety.

All adverse events (AEs) were recorded according to the Medical Dictionary for Regulatory Activities code, version 17.0 and World Health Organization Drug Dictionary (March 2014 version). Severity of AEs was described and graded using the National Cancer Institute Common Terminology Criteria for Adverse Events version 4.03.

Exploratory analyses included body weight, health‐related quality of life (HRQoL), pharmacokinetics, and biomarkers. Patients’ HRQoL was assessed using the Functional Assessment of Cancer Therapy‐Colorectal (FACT‐C) questionnaire (version 4).^15^

### 2.6 Statistical analysis

For part 1, based on the cohort size of 9, we estimated that the probability of observing DLT rates of ≥33.3% was 54% when the true toxicity rate was 30%. Sample size estimation for part 2 was based on the primary endpoint of OS. Final analyses were planned when 121 and 125 deaths occurred in the 2 combined treatment groups of substudies 1 and 2, respectively. For both substudies, the number of events above would provide 80% power to detect a hazard ratio (HR) of 0.6 based on a 2‐sided type 1 error of 0.05, an interim analysis (efficacy and futility for substudy 1; futility only for substudy 2) of OS at 50% of the total events. Within an 18‐month accrual period, 160 and 186 patients were planned to be randomized (1:1) in substudy 1 and substudy 2, respectively. An interim analysis was planned when approximately 61 and 63 deaths occurred in the 2 combined treatment groups in substudy 1 and substudy 2, respectively.

For both substudies in part 2, a stratified log‐rank test was used to analyze the OS and PFS differences between treatment groups. The HR and its 95% confidence interval (CI) was estimated based on the stratified Cox regression model using Efron's method accounting for ties. The analyses of OS and PFS were stratified by mGPS status (1 vs. 2) and geographical region (NA vs. ROW) in substudy 1, and by geographical region in substudy 2. ORR was compared between treatment groups in substudy 2 using Fisher's exact test. Subgroup analyses of OS by mGPS at baseline (1 vs. 2 [substudy 1 only]), geographic region (NA vs. ROW), ECOG PS at baseline (0 vs. 1; 0 vs. 2), gender (male vs. female), and age (≤65 y vs. >65 y) were performed. The HR and 95% CIs were provided for each subgroup comparison based on the Cox proportional hazards model.

The safety run‐in population included all patients enrolled in part 1 who received ≥1 dose of ruxolitinib or regorafenib. Data were summarized by assigned dose groups. For part 2, efficacy analyses were conducted on the intent‐to‐treat (ITT) population (all randomized patients); safety and tolerability analyses were conducted on the safety population (all randomized patients who received ≥1 dose of regorafenib, ruxolitinib, or placebo).

Body weight and change from baseline in body weight at scheduled assessment times were summarized. A linear, mixed, repeated‐measures model was used to model FACT‐C scale and subscale scores at the start of each cycle.

Analyses were performed using SAS version ≥9.0 (SAS Institute, Cary, North Carolina).

## 3 RESULTS

Between 17 March 2014 (enrollment of first patient) and 19 March 2016 (final data cutoff date), 11 and 396 patients were enrolled in part 1 and part 2 (substudy 1: n = 175; substudy 2: n = 221), respectively. Substudy 1 was terminated for futility (27 January 2016) based on a review of the interim analysis by the data monitoring committee. These results, together with results from an interim analysis from a phase 3 trial of ruxolitinib plus capecitabine that showed no additional benefit over capecitabine alone in patients with advanced pancreatic cancer and high systemic inflammation,^16^ led to the termination of substudy 2 (11 February 2016).

Enrollment was complete at the time of study termination. The number of events required for final efficacy analyses was reached in substudy 1 but not in substudy 2. Patients who were deemed by the investigator to be deriving benefit were allowed to continue treatment after discussion with the sponsor medical monitor.

### 3.1 Part 1: Patient Disposition, Exposure, and DLTs

In part 1, all 11 patients enrolled received ruxolitinib 20 mg BID plus regorafenib 160 mg QD. Median exposure was 110 and 105 days for ruxolitinib and regorafenib, respectively. Dose reduction was noted in 2 patients (18.2%) for ruxolitinib and 6 patients (54.5%) for regorafenib. All 11 patients discontinued treatment: 5 (45.5%) due to disease progression, 3  (27.3%) due to AEs, 1 (9.1%) due to death (cardiac arrest, unrelated to either study drug), and 2 (18.2%) for other reasons.

All patients experienced ≥1 treatment‐emergent adverse event (TEAE); all patients (except 1) experienced ≥1 grade 3/4 TEAE. The most common grade 3/4 nonhematologic TEAEs were palmar‐plantar erythrodysesthesia (PPE) syndrome (n = 3; 27.3%) and hypertension (n = 2; 18.2%). The most common grade 3/4 hematologic AEs (new/worsening laboratory abnormalities) were anemia (n = 4; 36.4%) and thrombocytopenia (n = 2; 18.2%). Seven patients (63.6%) experienced serious AEs (SAEs; abdominal pain, bacteremia, cardiac arrest, gastroenteritis, hyperbilirubinemia, perirectal abscess, pneumonia, pyrexia, sepsis, and small intestine obstruction [n = 1 each; 9.1%]).

One patient experienced a DLT of grade 3 PPE syndrome. Treatment was interrupted for 25 days, following which the toxicity resolved to grade 2 and the patient restarted treatment with a reduced dose of regorafenib (80 mg QD) with no change in the dose of ruxolitinib.

As <3 DLTs were reported in cycle 1 of part 1, per protocol, doses of ruxolitinib 15 mg BID and regorafenib 160 mg QD were selected for investigation in part 2, with titration to ruxolitinib 20 mg BID allowed in patients who tolerated the combination.

### 3.2 Part 2

#### 3.2.1 Patient baseline characteristics, disposition, and treatment exposure

In substudy 1, 87 and 88 patients were randomized into the ruxolitinib and placebo groups, respectively. In substudy 2, 110 and 111 patients were randomized into the ruxolitinib and placebo groups, respectively. Baseline characteristics were similar across groups in both substudies (Table 1).

**Table 1:** Patient Demographics and Baseline Characteristics (Intent‐To‐Treat Population)

Characteristics	Substudy 1 (n = 175)		Substudy 2 (n = 221)	
	Ruxolitinib+Regorafenib (n = 87)	Placebo+Regorafenib (n = 88)	Ruxolitinib+Regorafenib (n = 110)	Placebo+Regorafenib (n = 111)
Age, median (range), y	62.0 (34‐84)	60.0 (36‐81)	59.0 (37‐79)	61.0 (19‐83)
Sex, n (%)				
Male	53 (60.9)	56 (63.6)	62 (56.4)	57 (51.4)
Female	34 (39.1)	32 (36.4)	48 (43.6)	54 (48.6)
Race, n (%)				
White/Caucasian	70 (80.5)	70 (79.5)	84 (76.4)	80 (72.1)
Black/African American	7 (8.0)	7 (8.0)	6 (5.5)	7 (6.3)
Asian	0 (0.0)	2 (2.3)	10 (9.1)	9 (8.1)
Native Hawaiian/Pacific Islander	0 (0.0)	0 (0.0)	1 (0.9)	0 (0.0)
Other	10 (11.5)	9 (10.2)	5 (4.5)	11 (9.9)
Missing	0 (0.0)	0 (0.0)	4 (3.6)	4 (3.6)
mGPS, n (%)^a^				
0	3 (3.4)	1 (1.1)	103 (93.6)	99 (89.2)
1	66 (75.9)	69 (78.4)	2 (1.8)	3 (2.7)
2	16 (18.4)	17 (19.3)	2 (1.8)	4 (3.6)
Missing	2 (2.3)	1 (1.1)	3 (2.7)	5 (4.5)
ECOG performance status, n (%)				
0	27 (31.0)	21 (23.9)	53 (48.2)	40 (36.0)
1	52 (59.8)	62 (70.5)	54 (49.1)	64 (57.7)
2	6 (6.9)	5 (5.7)	2 (1.8)	5 (4.5)
Missing	2 (2.3)	0 (0.0)	1 (0.9)	2 (1.8)
Site of metastatic disease, n (%)				
Bone	14 (16.1)	6 (6.8)	8 (7.3)	8 (7.2)
Liver	67 (77.0)	71 (80.7)	80 (72.7)	74 (66.7)
Lung	67 (77.0)	59 (67.0)	84 (76.4)	68 (61.3)
Lymph nodes	28 (32.2)	36 (40.9)	31 (28.2)	30 (27.0)
Rectum	5 (5.7)	5 (5.7)	2 (1.8)	0 (0.0)
Ascites	6 (6.9)	2 (2.3)	1 (0.9)	6 (5.4)
Pleural effusion	2 (2.3)	2 (2.3)	1 (0.9)	2 (1.8)
Other	19 (21.8)	23 (26.1)	24 (21.8)	33 (29.7)
*KRAS* mutation status, n (%)				
No	36 (41.4)	31 (35.2)	42 (38.2)	49 (44.1)
Yes	48 (55.2)	51 (58.0)	63 (57.3)	58 (52.3)
Unknown	1 (1.1)	6 (6.8)	5 (4.5)	4 (3.6)
Missing	2 (2.3)	0 (0.0)	0 (0.0)	0 (0.0)
*BRAF* mutation status, n (%)				
No	38 (43.7)	31 (35.2)	42 (38.2)	45 (40.5)
Yes	2 (2.3)	3 (3.4)	3 (2.7)	6 (5.4)
Unknown	45 (51.7)	54 (61.4)	65 (59.1)	60 (54.1)
Missing	2 (2.3)	0 (0.0)	0 (0.0)	0 (0.0)
Prior systemic therapy	85 (97.7)	88 (100.0)	110 (100.0)	111 (100.0)
Prior systemic therapy regimens, n (%)				
1	0 (0.0)	1 (1.1)	3 (2.7)	3 (2.7)
2	11 (12.6)	13 (14.8)	18 (16.4)	21 (18.9)
3	20 (23.0)	20 (22.7)	32 (29.1)	24 (21.6)
4	20 (23.0)	16 (18.2)	19 (17.3)	29 (26.1)
≥5	34 (39.1)	38 (43.2)	38 (34.5)	34 (30.6)
Prior radiation therapy	27 (31.0)	32 (36.4)	44 (40.0)	45 (40.5)
Prior surgery or surgical procedure	66 (75.9)	79 (89.8)	93 (84.5)	99 (89.2)
Weight, kg				
Mean (SD)	78.2 (21.8)^b^	76.9 (19.5)	78.6 (20.0)	76.7 (19.1)^c^
C‐reactive protein, mg/L^d^				
Normal	3 (3.6)	1 (1.2)	78 (74.3)	69 (67.6)
High	81 (96.4)	85 (98.8)	27 (25.7)	33 (32.4)

ECOG, Eastern Cooperative Oncology Group; mGPS, modified Glasgow Prognostic Score.

a mGPS as collected on case report form.

b n = 85.

c n = 110.

d Substudy 1: ruxolitinib+regorafenib, n = 84; placebo+regorafenib, n = 86. Substudy 2: ruxolitinib+regorafenib, n = 105; placebo+regorafenib, n = 102.

**Figure 2:**
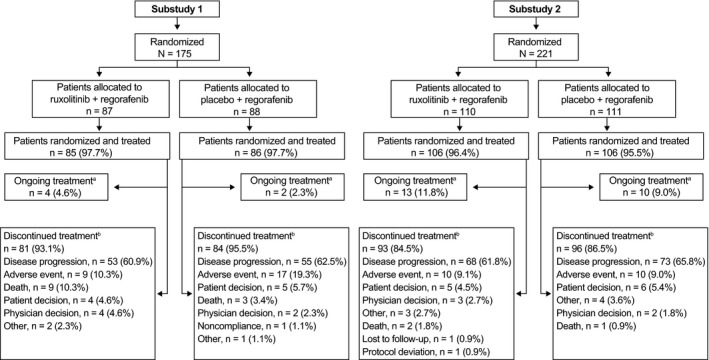
Patient disposition in substudy 1 and substudy 2. ^a^At the time of study termination (date of study termination: substudy 1, 27 January 2016; substudy 2, 11 February 2016). ^b^Before study termination.

Of 175 patients in substudy 1, 171 patients (97.7%) received treatment (Figure 2). For ruxolitinib and placebo, median exposure was 57 and 56 days, respectively. For regorafenib in the ruxolitinib and placebo groups, median exposure was 57 and 49 days, respectively. Treatment discontinuations were mainly due to disease progression or AEs (Figure 2). Six patients were still receiving study treatment at the time of study termination.

Of 221 patients in substudy 2, 212 (95.9%) received treatment (Figure 2). For ruxolitinib and placebo, median exposure was 106 and 56 days, respectively. For regorafenib in the ruxolitinib and placebo groups, median exposure was 103 and 49 days, respectively. Treatment discontinuations were mainly due to disease progression or AEs (Figure 2). Twenty‐three patients were still receiving study treatment at the time of study termination. Dose modifications are provided in the supplemental material (Table S1).

#### 3.2.2 Efficacy

**Figure 3:**
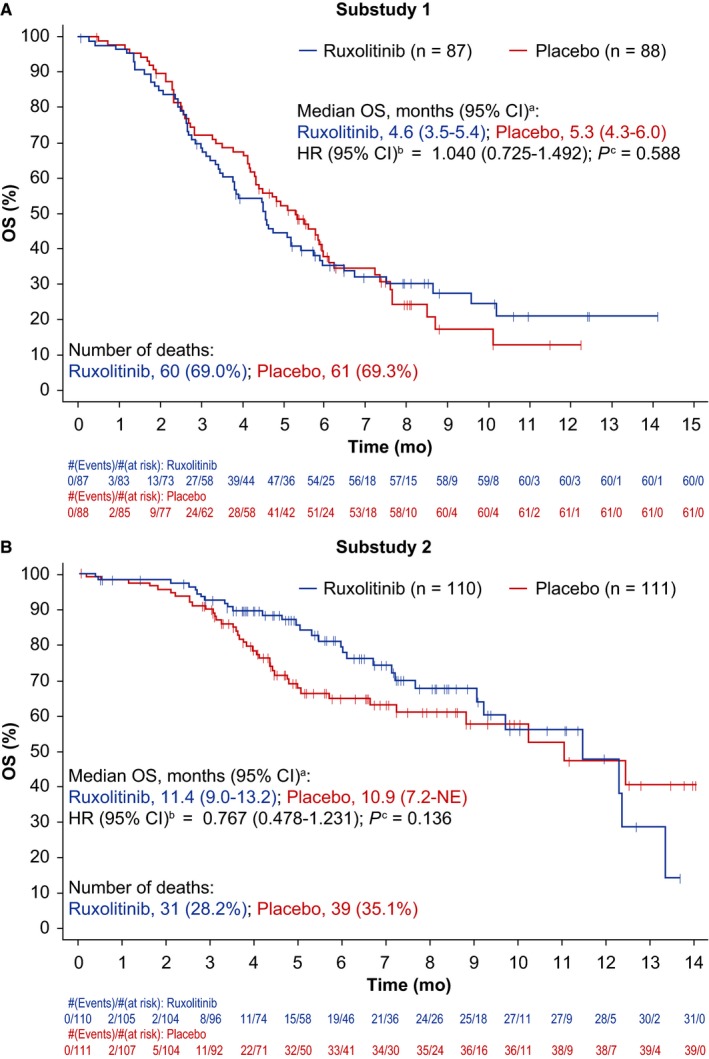
Overall survival Kaplan‐Meier plots of substudy 1 and substudy 2 (intent‐to‐treat population). ^a^Calculated using the method of Brookmeyer and Crowley (1982).^29^ ^b^Estimated using a Cox regression model with Efron's method used for ties, stratified by mGPS score and geographical region in substudy 1, and by geographical region in substudy 2. ^c^One‐sided *P* value calculated from log‐rank test stratified by mGPS score and geographical region in substudy 1, and by geographical region in substudy 2. Abbreviations: CI, confidence interval; HR, hazard ratio; mGPS, modified Glasgow Prognostic Score; NE, not estimable; OS, overall survival

**Figure 4:**
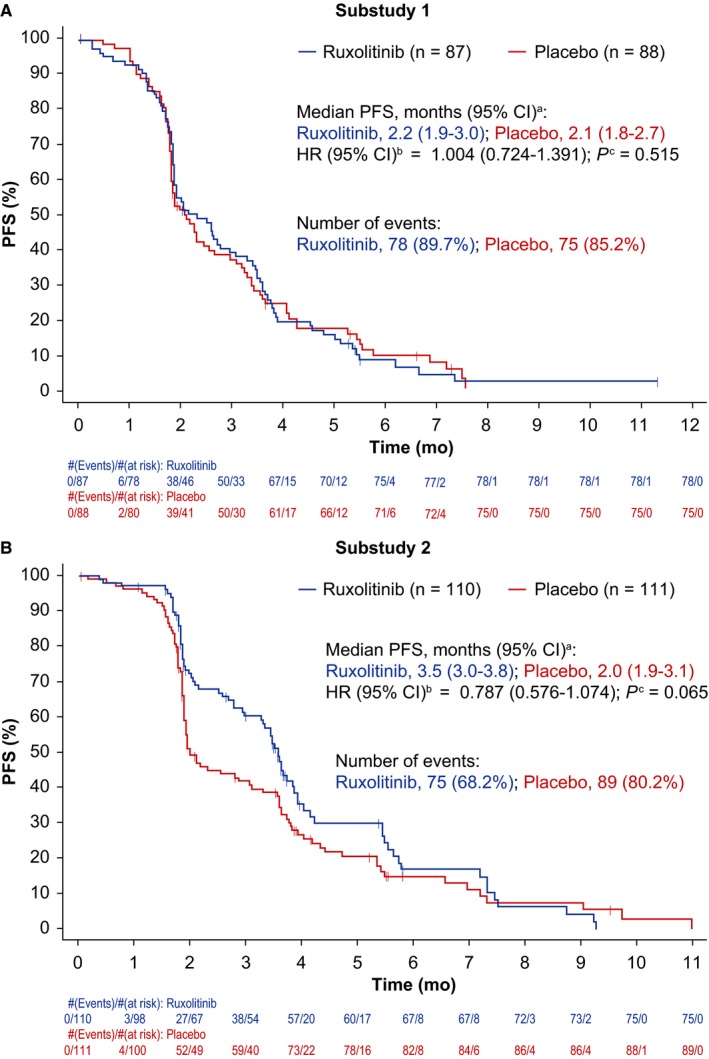
Progression‐free survival Kaplan‐Meier plots of substudy 1 and substudy 2 (intent‐to‐treat population). ^a^Calculated using the method of Brookmeyer and Crowley (1982).^29^ ^b^Estimated using a Cox regression model with Efron's method used for ties, stratified by mGPS score and geographical region in substudy 1, and by geographical region in substudy 2. ^c^One‐sided *P* value calculated from log‐rank test stratified by mGPS score and geographical region in substudy 1, and by geographical region in substudy 2. Abbreviations: CI, confidence interval; HR, hazard ratio; mGPS, modified Glasgow Prognostic Score; PFS, progression‐free survival

No statistically significant difference was observed between treatment groups of substudies 1 or 2 for the primary endpoint of OS (Figure 3A,B). Similarly, statistical significance was not achieved for PFS or ORR (Figure 4A,B; Table 2); however, there was a nonsignificant trend toward improved PFS in the ruxolitinib group compared with the placebo group in substudy 2 (Figure 4B). In subgroup analyses, OS was not significantly improved by ruxolitinib plus regorafenib vs. placebo plus regorafenib for any baseline factor tested, in either substudy 1 or 2 (Figure S1a,b).

**Table 2:** Overall Response Rate (Intent‐To‐Treat Population)

No (%)	Substudy 1 (n = 175)		Substudy 2 (n = 221)	
	Ruxolitinib + Regorafenib (n = 87)	Placebo + Regorafenib (n = 88)	Ruxolitinib + Regorafenib (n = 110)	Placebo + Regorafenib (n = 111)
Overall response rate (complete response + partial response) [95% CI]^a^	0 (0.0) [0‐4.2]	0 (0.0) [0‐4.1]	3^b^ (2.7) [0.6‐7.8]	5^b^ (4.5) [1.5‐10.2]
Stable disease	35 (40.2)	30 (34.1)	65 (59.1)	36 (32.4)
Progressive disease	32 (36.8)	39 (44.3)	30 (27.3)	56 (50.5)
Not evaluable	3 (3.4)	1 (1.1)	2 (1.8)	3 (2.7)
Not assessed	17 (19.5)	18 (20.5)	10 (9.1)	11 (9.9)

CI, confidence interval.

a Calculated based on the exact method for binomial distributions.

b Partial responses.

In exploratory analyses, no meaningful differences between treatment groups were observed in changes from baseline in body weight, HRQoL, FACT‐C score, or CRP levels (Data S1).

#### 3.2.3 Safety

All patients (except 1 in substudy 2, placebo group) experienced ≥1 TEAE (Table 3). The most common grade 3/4 nonhematologic TEAEs were PPE syndrome, hypertension, and abdominal pain (Table 4). The most common grade 3/4 hematologic AEs (new/worsening laboratory abnormalities) were anemia and lymphopenia (Table S2). SAEs occurring in ≥5 patients in any group of either substudy were abdominal pain, sepsis, pneumonia, dyspnea, and small intestinal obstruction. Twenty‐one and 6 patients experienced fatal TEAEs in substudies 1 and 2, respectively (Table 3); 1 fatal TEAE (suicide in substudy 1) was deemed related to ruxolitinib and regorafenib.

**Table 3:** Safety Summary of TEAEs

No (%)	Substudy 1 (n = 171)		Substudy 2 (n = 212)	
	Ruxolitinib + Regorafenib (n = 85)	Placebo + Regorafenib (n = 86)	Ruxolitinib + Regorafenib (n = 106)	Placebo + Regorafenib (n = 106)
Patients with any TEAEs	85 (100.0)	86 (100.0)	106 (100.0)	105 (99.1)
Patients with grade 3/4 TEAEs	70 (82.4)	70 (81.4)	82 (77.4)	77 (72.6)
Patients with any serious TEAEs	49 (57.6)	42 (48.8)	37 (34.9)	37 (34.9)
Patients with a fatal TEAE^a^	13 (15.3)	8 (9.3)	2 (1.9)	4 (3.8)
Patients who discontinued ruxolitinib/placebo because of TEAEs	12 (14.1)	17 (19.8)	12 (11.3)	11 (10.4)

TEAE, treatment‐emergent adverse event.

a Fatal TEAEs in substudy 1: general physical health deterioration (n = 3, ruxolitinib group; n = 2, placebo group); acute respiratory failure (n = 1 each group); disease progression (n = 2, ruxolitinib group); gastrointestinal hemorrhage, large intestinal obstruction, hepatic failure, sepsis, hepatic encephalopathy, obstructive pulmonary disease, and suicide (n = 1 each, ruxolitinib group); dyspnea (n = 2, placebo group); multi‐organ failure, obstruction, bile duct obstruction, hepatorenal syndrome, and malignant neoplasm progression (n = 1 each, placebo group). Fatal TEAEs in substudy 2: dyspnea (n = 1 each group); anemia and myocardial infarction (n = 1 each, ruxolitinib group); arrhythmia, coronary artery arteriosclerosis, abdominal distension, vomiting, and general physical health deterioration (n = 1 each, placebo group).

**Table 4:** Most Common (≥20% Events in Any Arm) Nonhematologic Treatment‐Emergent Adverse Events, All‐Grade

No (%)	Substudy 1 (n = 171)				Substudy 2 (n = 212)			
	Ruxolitinib+Regorafenib (n = 85)		Placebo+Regorafenib (n = 86)		Ruxolitinib+Regorafenib (n = 106)		Placebo+Regorafenib (n = 106)	
	All Grades	Grade 3/4	All Grades	Grade 3/4	All Grades	Grade 3/4	All Grades	Grade 3/4
PPE syndrome	36 (42.4)	11 (12.9)	38 (44.2)	13 (15.1)	61 (57.5)	18 (17.0)	50 (47.2)	14 (13.2)
Diarrhea	32 (37.6)	3 (3.5)	27 (31.4)	2 (2.3)	41 (38.7)	7 (6.6)	29 (27.4)	6 (5.7)
Decreased appetite	31 (36.5)	1 (1.2)	31 (36.0)	2 (2.3)	28 (26.4)	1 (0.9)	35 (33.0)	3 (2.8)
Fatigue	29 (34.1)	3 (3.5)	31 (36.0)	5 (5.8)	43 (40.6)	5 (4.7)	47 (44.3)	10 (9.4)
Abdominal pain	27 (31.8)	12 (14.1)	31 (36.0)	9 (10.5)	28 (26.4)	6 (5.7)	31 (29.2)	8 (7.5)
Constipation	27 (31.8)	0 (0.0)	21 (24.4)	2 (2.3)	24 (22.6)	1 (0.9)	22 (20.8)	0 (0.0)
Nausea	22 (25.9)	2 (2.4)	22 (25.6)	2 (2.3)	30 (28.3)	0 (0.0)	22 (20.8)	4 (3.8)
Asthenia	22 (25.9)	4 (4.7)	22 (25.6)	5 (5.8)	19 (17.9)	3 (2.8)	23 (21.7)	6 (5.7)
Stomatitis	21 (24.7)	4 (4.7)	18 (20.9)	3 (3.5)	19 (17.9)	1 (0.9)	21 (19.8)	2 (1.9)
Hypertension	18 (21.2)	8 (9.4)	22 (25.6)	6 (7.0)	44 (41.5)	23 (21.7)	42 (39.6)	17 (16.0)
Vomiting	16 (18.8)	3 (3.5)	20 (23.3)	2 (2.3)	24 (22.6)	2 (1.9)	15 (14.2)	6 (5.7)
Headache	13 (15.3)	1 (1.2)	16 (18.6)	1 (1.2)	21 (19.8)	1 (0.9)	26 (24.5)	0 (0.0)
Dysphonia	11 (12.9)	0 (0.0)	14 (16.3)	0 (0.0)	22 (20.8)	0 (0.0)	27 (25.5)	0 (0.0)

PPE, palmar‐plantar erythrodysesthesia.

## 4 DISCUSSION

In this study in patients with relapsed/refractory mCRC, ruxolitinib combined with regorafenib did not show increased safety concerns; however, no survival benefit was observed in the ruxolitinib vs. the placebo group. Results from this study suggest that further investigation of ruxolitinib combined with regorafenib in mCRC is not warranted.

Several preclinical studies suggested the use of selective JAK1/2 inhibitors as potential therapy for treatment of CRC, thereby, providing a rationale for randomized controlled trials.^17^, ^18^ Also, a subgroup analysis of the randomized, phase 2 RECAP study suggested a survival benefit with ruxolitinib in combination with capecitabine vs. capecitabine alone, in patients with metastatic pancreatic cancer and high CRP levels.^9^ Altogether, these results suggested a potential benefit of JAK inhibition in solid tumors. However, 2 phase 3 studies (JANUS 1 and JANUS 2) that assessed the efficacy and safety of ruxolitinib plus capecitabine in patients with metastatic pancreatic cancer requiring second‐line therapy were prematurely terminated based on an interim futility analysis.^16^ Furthermore, data from the present randomized study do not support the utility of ruxolitinib in patients with relapsed/refractory mCRC regardless of levels of inflammation. Whereas it is possible that ruxolitinib might offer greater benefit if introduced earlier in the course of CRC, a study designed to assess this could be confounded by the larger number of available treatment options and the pronounced disease heterogeneity in early stage CRC vs. late stage CRC.^19^ Such a study would also require a larger sample size than that of the current study. Moreover, early administration of ruxolitinib in these patients receiving first‐line therapies for mCRC (such as fluorouracil, leucovorin, in combination with irinotecan [FOLFIRI] or oxaliplatin [FOLFOX]) would be challenging given that myelosuppression is associated with all of these treatments.^20^, ^21^

With or without ruxolitinib, median OS in substudy 1 (4.6 and 5.3 months, respectively) was slightly lower than that observed in the phase 3 CORRECT study evaluating regorafenib vs. placebo in patients with refractory mCRC (6.4 months with regorafenib).^11^ However, compared with substudy 1, the median OS in substudy 2 was approximately twice as long (11.4 and 10.9 months in the ruxolitinib and placebo groups, respectively). The reason for the overall prolonged OS in substudy 2 vs. substudy 1 could be the lower levels of systemic inflammation (ie, mGPS 0 vs. 1/2, respectively). This is in line with a review of data from over 30 000 patients with different tumor types across 60 studies, which showed that chronic systemic inflammatory response, as evidenced by high GPS/mGPS, is clearly associated with poorer prognosis.^14^ As no systemic inflammation‐based stratification was performed in the CORRECT study, this may explain why patients treated with regorafenib in the CORRECT study had longer median OS than those in substudy 1, but shorter than those in substudy 2. These results underscore the importance of further identification of biomarkers (eg, other determinants of inflammation such as thrombocytosis or lymphocyte/neutrophil ratio) and patient clinical characteristics that could predict for benefit in mCRC, to improve patient selection for future treatment strategies. For example, a cohort study of regorafenib use in clinical practice in patients with refractory mCRC (the REBECCA study) showed that a high ECOG PS (≥2), a shorter time from initial diagnosis of metastases (<18 months), an initial regorafenib dose <160 mg, >3 metastatic sites, liver metastases, and *KRAS* mutations were independently associated with poorer survival.^22^

In addition to the key role of systemic inflammation and JAK/STAT pathway activation in various solid tumors, evidence suggests that clinical responses to chemotherapy can be improved if immunogenic cell death pathways are concurrently activated.^23^ A preclinical study of an immuno‐competent syngeneic PAN02 pancreatic model showed that combining JAK inhibitors with other immunomodulatory agents inhibiting indoleamine‐pyrrole 2,3‐dioxygenase 1 (IDO1), phosphatidylinositol‐3 kinase delta (PI3Kδ), or programmed death‐1/programmed death‐ligand 1 (PD‐1/PD‐L1) resulted in enhanced antitumor activity.^24^ Therefore, there may yet be value in combining JAK inhibiors with chemotherapy and/or immune response modifiers. Given the persistent importance of JAK/STAT signaling pathway in tumorigenesis in various cancers, including colorectal cancer, it is possible that simultaneous targeting of other regulators involved in this complex pathway and the optimization of combination therapy regimens may hold potential to achieve improved outcomes.

Overall, the safety profile of ruxolitinib 15‐20 mg BID in combination with regorafenib in patients with relapsed/refractory mCRC was acceptable and was consistent with that reported in previous trials of ruxolitinib in other tumors.^9^, ^25^, ^26^, ^27^, ^28^ No new safety concerns were reported for either treatment groups in either substudy.

In conclusion, results from our study do not support the use of the combination of ruxolitinib with regorafenib in patients with relapsed/refractory mCRC.

## CONFLICT OF INTEREST

D. Fogelman and J. Bendell received research funding from Incyte. P. García‐Alfonso received honoraria and served as a consultant for Amgen, Merck, Roche, Sanofi, and Servier. J. Nemunaitis is an employee and stockholder of Gradalis Inc; received honoraria, served as a consultant, and served on speakers bureau for Amgen. J. M. Vieitez received honoraria and served as a consultant for Amgen, Merck, and Roche, and served on speakers bureau for Roche. A. Cohn received honoraria and served on speakers bureau for Medimmune and Taiho. G. Saylors served on speakers bureau for Merck. A. Assad, J. Switzky, and L. Zhou are employees and stockholders of Incyte Corporation. A. Cubillo, M. L. L. Mirón, D. Flora, C. Borg, and L. Mineur have nothing to disclose.
