# Supplementary material for: Randomized, double‐blind, phase two study of ruxolitinib plus regorafenib in patients with relapsed/refractory metastatic colorectal cancer
Source: Cancer Med. 2018 Aug 19;7(11):5382–93. doi: 10.1002/cam4.1703 (PMC6246927; doi:10.1002/cam4.1703)

**Supplementary Material**

**Randomized, Double-blind, Phase 2 Study of Ruxolitinib Plus Regorafenib in Patients With Relapsed/Refractory Metastatic Colorectal Cancer**David Fogelman, Antonio Cubillo, Pilar García-Alfonso, María Luisa Limón Mirón, John Nemunaitis, Daniel Flora, Christophe Borg, Laurent Mineur, Jose M. Vieitez, Allen Cohn,
Gene Saylors, Albert Assad, Julie Switzky, Li Zhou, Johanna Bendell

***Exploratory Analyses Results***

The mean (SD) percentage change in body weight from baseline to the end of treatment in substudy 1 was −4.2 (6.0) with ruxolitinib (n=44) and −5.7 (5.0) with placebo (n=51), and in substudy 2 was −2.5 (4.8) with ruxolitinib (n=68) and −4.3 (5.7) with placebo (n=77). In substudy 1, the mean (SD) baseline FACT-C total score was 98.5 (16.1) in the ruxolitinib group and 91.2 (21.4) in the placebo group; the mean (SD) best postbaseline percentage change from baseline was 0.9 (11.8) in the ruxolitinib group and −4.0 (17.2) in the placebo group. In substudy 2, the mean (SD) baseline FACT-C total score was 104.4 (18.1) in the ruxolitinib group and 98.7 (20.3) in the placebo group; the mean (SD) best postbaseline percentage change from baseline was 1.7 (12.9) in the ruxolitinib group and −3.2 (12.9) in the placebo group. At the end of treatment, ≥50% of patients in both substudies and in both treatment groups had high CRP levels (substudy 1: ruxolitinib, 94.4% [34/36 patients]; placebo, 95.5% [42/44 patients]; substudy 2: ruxolitinib, 74.1% [40/54 patients]; placebo, 68.9% [42/61 patients]). Due to study termination, pharmacokinetic analyses were not performed.

SUPPLEMENTAL TABLES

**TABLE S1.** Dose modifications in substudy 1 and substudy 2

| No (%) | Substudy 1 (n=171) | | | Substudy 2 (n=212) | | | |  |
| --- | --- | --- | --- | --- | --- | --- | --- | --- |
|  | Ruxolitinib+ Regorafenib  (n=85) | Placebo+ Regorafenib  (n=86) | | Ruxolitinib+ Regorafenib  (n=106) | | Placebo+ Regorafenib  (n=106) | |  |
| Ruxolitinib/Placebo |  | |  | |  | |  | |
| Dose interruption | 24 (28.2) | | 25 (29.1) | | 34 (32.1) | | 40 (37.7) | |
| Dose reduction | 4 (4.7) | | 5 (5.8) | | 7 (6.6) | | 8 (7.5) | |
| Dose escalation | 10 (11.8) | | 5 (5.8) | | 9 (8.5) | | 6 (5.7) | |
| Regorafenib |  | |  | |  | |  | |
| Dose interruption | 40 (47.1) | | 49 (57.0) | | 55 (51.9) | | 57 (53.8) | |
| Dose reduction | 30 (35.3) | | 41 (47.7) | | 62 (58.5) | | 54 (50.9) | |
| Dose escalation | 1 (1.2) | | 1 (1.2) | | 5 (4.7) | | 4 (3.8) | |

**TABLE S2.** Worsening of hematologic toxicity^a^

| No (%) | Substudy 1 (n=171) | | | | Substudy 2 (n=212) | | | | |
| --- | --- | --- | --- | --- | --- | --- | --- | --- | --- |
|  | Ruxolitinib+ Regorafenib  (n=85) | | Placebo+ Regorafenib  (n=86) | | Ruxolitinib+ Regorafenib  (n=106) | | Placebo+ Regorafenib  (n=106) | | |
|  | All Grades | Grade 3/4 | All Grades | Grade 3/4 | All Grades | Grade 3/4 | All Grades | Grade 3/4 | |
| Anemia | 37 (43.5) | 3 (3.5) | 31 (36.0) | 4 (4.7) | 67 (63.2) | 9 (8.5) | 38 (35.8) | | 4 (3.8) |
| Lymphopenia | 31 (36.5) | 11 (12.9) | 31 (36.0) | 7 (8.1) | 51 (48.1) | 15 (14.2) | 41 (38.7) | | 7 (6.6) |
| Thrombocytopenia | 16 (18.8) | 1 (1.2) | 20 (23.3) | 1 (1.2) | 27 (25.5) | 1 (0.9) | 36 (34.0) | | 1 (0.9) |
| Leukopenia | 15 (17.6) | 0 (0.0) | 13 (15.1) | 0 (0.0) | 28 (26.4) | 1 (0.9) | 24 (22.6) | | 4 (3.8) |
| Neutropenia | 6 (7.1) | 0 (0.0) | 5 (5.8) | 1 (1.2) | 18 (17.0) | 3 (2.8) | 18 (17.0) | | 3 (2.8) |

^a^ Laboratory abnormalities.

SUPPLEMENTAL FIGURES

**FIGURE S1. Forest plot of overall survival by subgroup (intent-to-treat population)**

^a^ The overall HR was estimated using a Cox regression model with Efron’s method for ties, stratified by mGPS (Substudy 1 only) and Region. Abbreviations: CI, confidence interval; ECOG, Eastern Cooperative Oncology Group; HR, hazard ratio; mGPS, modified Glasgow Prognostic Score.


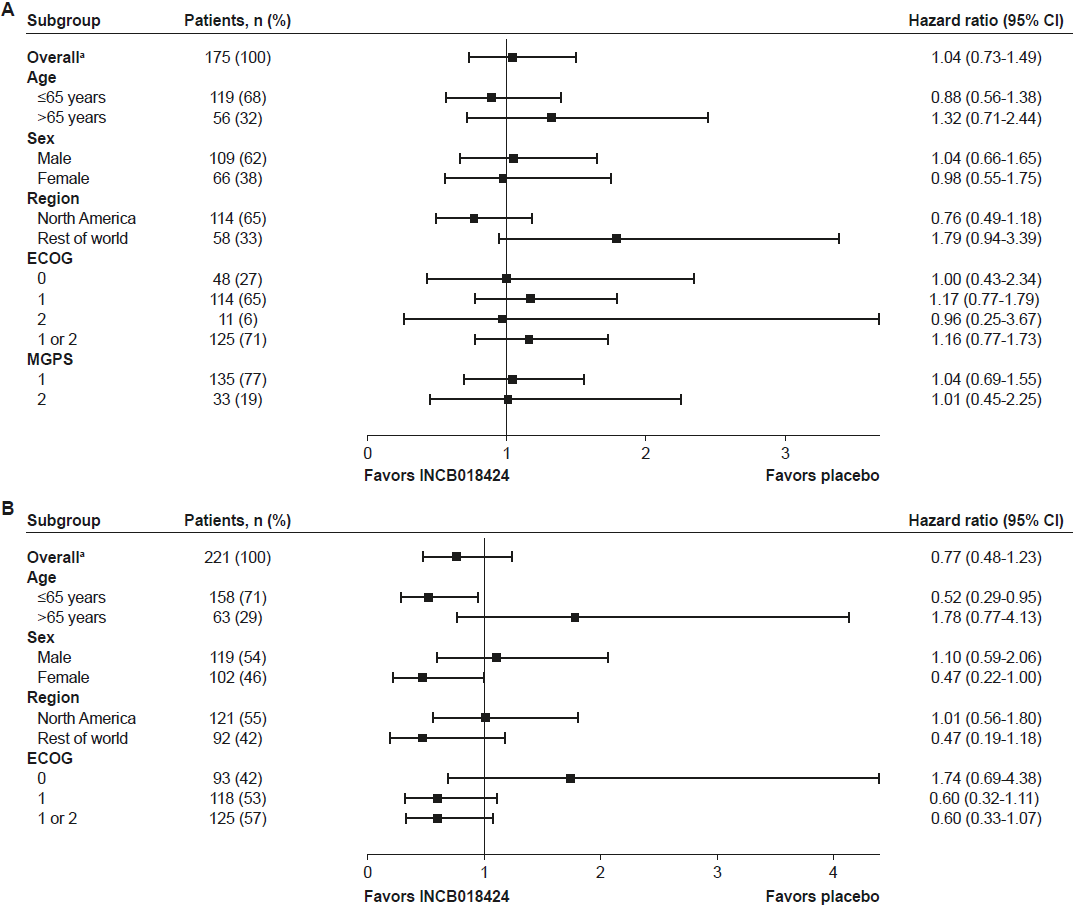

Supplement: Supplementary file 1 [file CAM4-7-5382-s001.docx]
